# Unraveling Microviscosity Changes Induced in Cancer Cells by Photodynamic Therapy with Targeted Genetically Encoded Photosensitizer

**DOI:** 10.3390/biomedicines12112550

**Published:** 2024-11-08

**Authors:** Liubov E. Shimolina, Aleksandra E. Khlynova, Vadim V. Elagin, Pavel A. Bureev, Petr S. Sherin, Marina K. Kuimova, Marina V. Shirmanova

**Affiliations:** 1 Institute of Experimental Oncology and Biomedical Technologies, Privolzhsky Research Medical University, Minin and Pozharsky Square, 10/1, 603005 Nizhny Novgorod, Russia; shimolina_l@pimunn.net (L.E.S.); khlynova_a@pimunn.net (A.E.K.); elagin_v@pimunn.net (V.V.E.); bureev_p@pimunn.net (P.A.B.); 2 Department of Chemistry, Imperial College London, White City Campus, London W12 0BZ, UK; p.sherin@imperial.ac.uk (P.S.S.); m.kuimova@imperial.ac.uk (M.K.K.)

**Keywords:** plasma membrane, cancer cell, microviscosity, molecular rotors, fluorescence lifetime imaging microscopy FLIM, genetically encoded photosensitizer, photodynamic therapy

## Abstract

Background: Despite the fundamental importance of cell membrane microviscosity, changes in this biophysical parameter of membranes during photodynamic therapy (PDT) have not been fully understood. Methods: In this work, changes in the microviscosity of membranes of live HeLa Kyoto tumor cells were studied during PDT with KillerRed, a genetically encoded photosensitizer, in different cellular localizations. Membrane microviscosity was visualized using fluorescence lifetime imaging microscopy (FLIM) with a viscosity-sensitive BODIPY2 rotor. Results: Depending on the localization of the phototoxic protein, different effects on membrane microviscosity were observed. With nuclear localization of KillerRed, a gradual decrease in microviscosity was detected throughout the entire observation period, while for membrane localization of KillerRed, a dramatic increase in microviscosity was observed in the first minutes after PDT, and then a significant decrease at later stages of monitoring. The obtained data on cell monolayers are in good agreement with the data obtained for 3D tumor spheroids. Conclusions: These results indicate the involvement of membrane microviscosity in the response of tumor cells to PDT, which strongly depends on the localization of reactive oxygen species attack via targeting of a genetically encoded photosensitizer.

## 1. Introduction

Photodynamic therapy (PDT) is a treatment method used in clinics to address cancerous and pre-cancerous lesions [1]. The principle of PDT is a photochemical reaction between a photosensitizer and oxygen or biomolecules initiated by the absorption light, which results in the generation of reactive oxygen species (ROS). The predominant mechanism of this reaction for most photosensitizers is energy transfer from a triplet state to molecular oxygen with the formation of singlet oxygen (^1^O_2_), referred to as type II photoreactions. Another type of photoreaction, type I, relies on electron or hydrogen atom transfer between a photosensitizer and a substrate with the formation of free radicals that further react with oxygen [2]. The type of photosensitized reaction depends on many factors, such as the nature and concentration of the sensitizer, oxygen content, and substrate reactivity. In a complex biological environment, both reactions can proceed in parallel.

Due to the high reactivity and short half-life of ROS, photodamage within a cell and the subsequent biological response strongly depend on the subcellular localization of the photosensitizer [3]. Chemical photosensitizers are typically distributed in multiple sites, including mitochondria, lysosomes, ER and the Golgi apparatus, and plasma membrane [4]. In these structures, membrane lipids are more vulnerable to oxidation during PDT due to the presence of double bonds in the fatty acid tails. Lipid oxidation causes marked changes in the physicochemical properties of membranes, such as permeability, polarity, lipid order, and microviscosity. While the effects of lipid oxidation on the membrane state are relatively well investigated in model lipid bilayers, there is very limited information about the changes in cellular membranes.

Fluorescence lifetime imaging microscopy (FLIM) using fluorescent molecular rotors has proven to be a reliable method to obtain micrometer-scale quantitative maps of viscosity in live cells and even tissues and monitor it in space and time [5]. Sensing of microviscosity with such probes is based on their ability for intramolecular twisting in the excited state, which is constrained in a more viscous environment, so that emission quantum yield and decay time increase. Among molecular rotors, BODIPY (boron-dipyrromethene) derivatives are considered highly effective viscosity probes due to their excellent photophysical properties, such as high quantum yields, narrow emission bands, a wide dynamic range of viscosity values that are measurable, and monoexponential time-resolved decays allowing easy interpretation of the data [6].

Molecular rotor detection has been used to monitor a large increase in membrane viscosity in live cells and model membranes due to lipid peroxidation in type II photoreactions [7,8,9,10]. The proposed mechanism for this change was the oxidation of unsaturated bonds in lipids by singlet oxygen at the site of irradiation and the subsequent formation of long-lived peroxide species that can travel further in lipid bilayers [7]. At the same time, a decrease in local viscosity was detected following type I photoreactions [7,8]. One of the possible mechanisms is the oxidative cleavage of C=C bonds in lipids, which results in their very loose packing and pore formation [11]. These trends were determined by using specific photosensitizers that are known to produce either radical species (e.g., methylene blue) or singlet molecular oxygen (porphyrins), and the mechanism of changes was established in model membranes that were assembled from synthetic oxidized/truncated lipids. However, the links between the localization of a photosensitizer within a cell and the type of photosensitized reactions were never explored. A significant challenge in this area is the targeted delivery of the same photosensitizer to different organelles in cells to enable a direct comparison.

We set out to study how the differential localization of the sensitizer affects ROS production and the effects. The only possible way to do this unambiguously is with a genetically encoded sensitizer. We used a genetically encoded photosensitizer, KillerRed, to induce a photodynamic reaction in a specific cellular compartment. This was followed by FLIM with molecular rotor BODIPY2 to dynamically track changes in the microviscosity of plasma membranes of cancer cells during PDT induced in different cellular localizations. Specifically, we compared the effects for the two localizations—(i) KillerRed within the plasma membranes (colocalized with BODIPY2 rotor) and (ii) within nuclei. There are not many such photosensitizers known, and among them, KillerRed is one of the most extensively studied. In addition, the choice of KillerRed was due to its spectral characteristics, which allow for multiparametric analysis with a molecular rotor that fluoresces in the green range. Another well-known genetically encoded photosensitizer, miniSOG, fluoresces in the blue region of the spectrum, which complicates its use in combination with a BODIPY molecular rotor [12].

KillerRed is a fluorescent protein of the class of green fluorescent proteins (GFPs) that possesses notable phototoxic properties, efficiently generating ROS via type I photoreactions [13,14]. The cause of its phototoxicity is the presence of a water-filled channel reaching the chromophore area from the end cap of the β-barrel and of two amino acid residues (Glu68 and Ser119) adjacent to the chromophore [14]. This combination is hypothesized to trigger photoinduced reduction in the acylimine group of the chromophore with subsequent abstraction of the electron by molecular oxygen. Although KillerRed is inferior in its phototoxicity to clinically approved porphyrin-based chemical photosensitizers, it represents a unique tool to induce a precise site of photodamage within a cell. As such, it has found applications for targeted protein inactivation, induction of oxidative stress in specified organelles, and cancer cell killing in preclinical settings [15,16]. While KillerRed has been used for PDT previously, its use was not combined with ROS monitoring in general or molecular rotor-based monitoring in particular, which presents a significantly novel result.

This study was carried out on monolayer HeLa Kyoto (human cervical cancer) cultures and in three-dimensional tumor spheroids stably expressing KillerRed within the plasma membrane or cellular nuclei. Photobleaching studies of KillerRed, cell viability, and ROS analyses were performed to validate the efficacy of PDT. The microviscosity of plasma membranes was monitored using FLIM and the fluorescent molecular rotor BODIPY2 localized in the hydrophobic lipid tail region of a lipid bilayer, which was previously used both in cell monolayers and in spheroids [17].

## 2. Materials and Methods

### 2.1. Cell Cultures and Generation of 3D Tumor Spheroids

**Figure 1:**
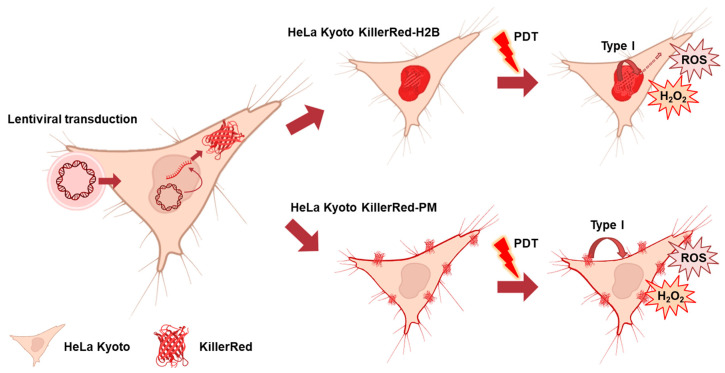
The schematic mechanism of action of KillerRed protein as a genetically encoded photosensitizer for PDT. The gene encoding KillerRed is incorporated in cancer cells (HeLa Kyoto) through lentiviral transduction to ensure the expression of the protein in a targeted compartment—the cell nucleus (KillerRed-H2B) or the plasma membrane (KillerRed-PM). PDT with KillerRed results in the formation of ROS via the type I photoreaction, which leads to oxidative damage in the targeting site and other compartments.

HeLa Kyoto (human cervical cancer) cells with the phototoxic protein KillerRed fused to histone H2B (H2B) and plasma membrane (PM) were used. The cell lines stably expressing KillerRed in these localizations have previously been obtained by lentiviral transduction [18]. The mechanism of action of KillerRed as a genetically encoded photosensitizer is schematically depicted in Figure 1. The cells were cultured in DMEM (Life Technologies, Carlsbad, CA, USA) containing 100 μg/mL penicillin, 100 μg/mL streptomycin sulfate, 2 mM L-glutamine, and 10% fetal bovine serum (FBS) at 37 °C in a humidified atmosphere with 5% CO_2_.

For PDT, the cells were seeded on 96-well plates with a glass bottom and black walls (SPL LifeSciences, Pocheon, Republic of Korea) in complete DMEM without phenol red (Life Technologies, Carlsbad, CA, USA). The complete medium was replaced with serum-free DMEM and irradiation was performed. A continuous laser with a wavelength of 593 nm MGL-III-593 (CNI, Qingdao, China) was used to irradiate HeLa Kyoto cells expressing the KillerRed protein. Laser radiation was directed perpendicularly to the plate surface. The laser power was controlled before each irradiation using a PM100A power meter (Thorlabs, Dortmund, Germany). The light spot diameter was 10 mm. The laser power was 50 mW/cm^2^. The cells were irradiated for 25 min, which corresponds to an energy density of 75 J/cm^2^.

To obtain spheroids, cells were seeded in 96-well round-bottomed, ultralow-attachment plates at a concentration of 100 cells in 200 μL of medium. Densely structured spheroids of ~300 μm in size were generated after 7 days.

For PDT, 7-day-old spheroids were carefully transferred to dark-walled 96-well plates (SPL LifeSciences, Republic of Korea) with 3–5 spheroids per well in DMEM without phenol red (Life Technologies, Carlsbad, CA, USA). PDT was performed using an MGL-III-593 laser (CNI, China) at a wavelength of 593 nm. The laser power was controlled before each irradiation using a PM100A power meter (Thorlabs, Germany). The intensity was 50 mW/cm^2^, the exposure time was 25 min, and the light dose was 75 J/cm^2^. The 3D spheroids were treated independently at each time point. Unirradiated spheroids served as controls.

### 2.2. Viscosity Imaging by FLIM with Molecular Rotor

**Figure 2:**
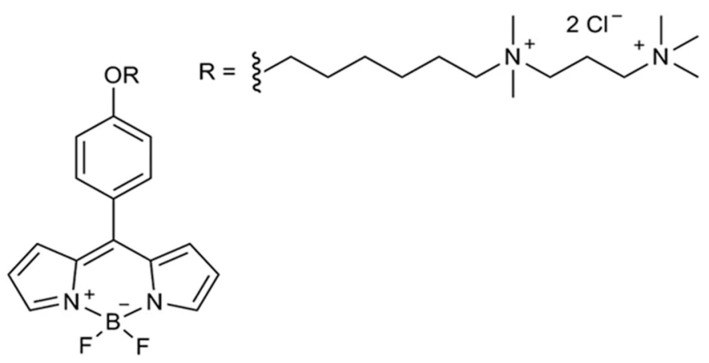
The molecular structure of molecular rotor BODIPY2.

Previously, we developed and tested protocols for staining the plasma membrane of cell monolayers and tumor spheroids [17]. The first step of staining was replacing the culture medium in the wells with ice-cold Hanks’s solution without Ca^2+^/Mg^2+^ to slow endocytosis. The cells were incubated at 4 °C for 3–5 min. Then Hanks’s solution was replaced with ice-cold BODIPY2 in PBS (4.5 μM for monolayer, 8.9 μM for spheroids). FLIM images were acquired for ~30 min after staining the cells with BODIPY2. The molecular structure of molecular rotor BODIPY2 is presented in Figure 2.

For viscous imaging, a LSM 880 laser scanning microscope (Carl Zeiss, Gottingen, Germany) equipped with a FLIM SPC 150 TCSPC module (Becker & Hickl GmbH, Berlin, Germany) and a Mai Tai HP femtosecond laser (80 MHz, 140 fs, Spectra Physics, Milpitas, CA, USA) were used. The excitation wavelength of BODIPY2 fluorescence in the two-photon mode was 850 nm, and the signal was detected in a range of 500 to 550 nm using filters. A C Plan-Apochromat 40×/1.3 NA objective was used for imaging. FLIM images were acquired at a laser power of ~6–8 mW and photon collection time of 60 s to ensure ≥5000 photons per decay curve using a binning factor of 1. Ten randomly selected fields of view were collected in each well.

SPCImage 8.3 software (Becker & Hickl GmbH, Germany) was used for molecular rotor fluorescence lifetime analysis in cell membranes. Fluorescence lifetime was analyzed in the plasma membrane of cultured cells by manual selection of regions of interest. BODIPY2 fluorescence decay curves were fitted to a monoexponential decay model. The goodness of fit (χ^2^ value) ranged from 0.8 to 1.2. Fluorescence lifetimes were converted to viscosity values using a calibration curve obtained previously [17].

### 2.3. Cell Viability Analysis

Live/dead cell counts were assessed using a live/dead dual-staining kit (Sigma, St. Louis, MO, USA). Calcein AM and propidium iodide (PI) staining was performed according to the manufacturer’s protocol. Monolayer cells were stained after 10 min, 1 h and 24 h, and spheroids were stained after 3 h and 6 h. Fluorescence images were obtained using an LSM 880 laser scanning microscope (Carl Zeiss, Gottingen, Germany). The calcein fluorescence excitation wavelength was 488 nm, and detection was in the range of 500–570 nm. The PI fluorescence excitation wavelength was 543 nm, and detection was in the range of 600–700 nm. The percentage of dead cells (stained with PI) from the total number of cells was calculated for quantitative assessment of live/dead cells.

### 2.4. Reactive Oxygen Species Assay

For reactive oxygen species analysis, 2′,7′-dichlorofluorescein diacetate (DCFH-DA) (Sigma, USA) was used. The cells were incubated with the dye at a concentration of 1 μM for 15 min in an incubator in a darkened box. Then, they were washed with PBS and imaging was performed. The cells were stained after 10 min and 1 h. Fluorescence images were obtained using an LSM 880 laser scanning microscope (Carl Zeiss, Gottingen, Germany). Probe fluorescence was excited using an argon laser at a wavelength of 488 nm, and fluorescence was recorded in the range of 500–570 nm.

### 2.5. Products of Lipid Peroxidation Assay

To detect diene and triene conjugates and Schiff bases, 0.1 mL of the control sample or after PDT was taken and 5 mL of a heptane–isopropanol mixture in a ratio of 3:7 was added to it. The resulting mixture was shaken for 15 min. After that, 1 mL of 0.01 M aqueous hydrochloric acid solution was added for phase separation. After phase separation, the upper heptane phase was transferred to a separate test tube, and 1 g of sodium chloride was added to the lower one to dehydrate the isopropanol extract, which was transferred to a clean test tube. For quantitative assessment, the transmittance was measured on a spectrophotometer. Each phase was evaluated against the control at wavelengths of 220, 232, 278, and 400 nm. The content of diene and triene conjugates and Schiff bases was estimated by the ratio of the transmittances at 232 nm (for diene conjugates), 278 nm (for triene conjugates), and 400 nm (for Schiff bases) to the transmittance at 220 nm (isolated double bonds). The obtained relative content of the products is presented as percentage of the negative control.

### 2.6. MTT Assay

For viability analysis, cells were seeded in 96-well plates (10 × 10^3^ cells/well) and incubated for 24 h. Then, PDT was performed. After 24 h, cells were treated with MTT (3(4,5-dimethyl-2-thiazolyl)-2,5-diphenyl-2*H*-tetrazolebromide) reagent (PanEco, Moscow, Russia) according to the manufacturer’s protocol and colorimetric analysis was performed at 570 nm using a multi-mode microplate reader (Synergy Mx; BioTek Instruments, Winooski, VT, USA). Cell viability was calculated as a percentage of untreated control cells. Three independent experiments with 8–10 internal replicates were performed for each cell line.

### 2.7. Statistical Analysis

All experiments were performed in triplicate, the results from which were then combined. Data are expressed as means ± SD. To estimate the statistical significance of the differences, ANOVA with Bonferroni’s post hoc test or a two-tailed Student’s *t*-test were used where appropriate (*p* < 0.05 was considered statistically significant).

## 3. Results

### 3.1. Cell Viability and ROS After PDT with KillerRed

**Figure 3:**
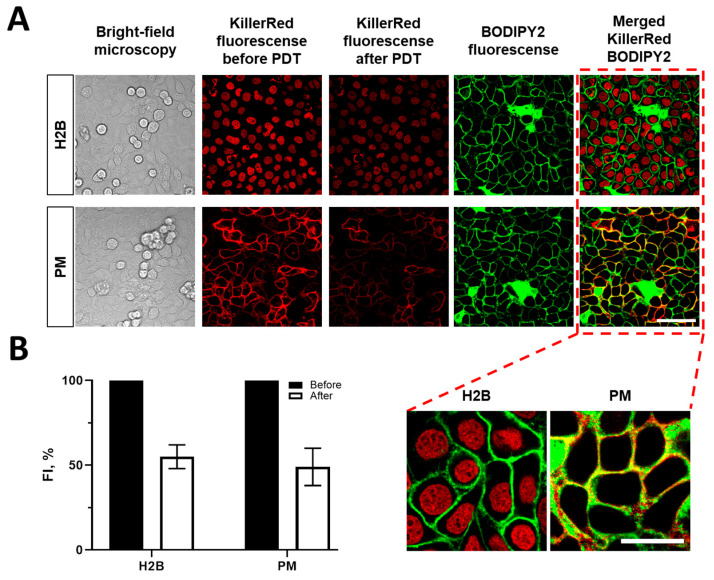
(**A**) Localization of the genetically encoded photosensitizer KillerRed in the nucleus and plasma membrane of HeLa cells and of the fluorescent molecular rotor in the plasma membrane. The scale bar is 40 µm, applicable to all images. (**B**) Photobleaching of KillerRed after PDT. Quantification of the fluorescence intensity in the cells after PDT. Means ± SD, *n* = 50 cells. The scale bar is 40 µm, applicable to all images. H2B: cells with nuclear localization of KillerRed, PM: cells with membrane localization of KillerRed.

To induce oxidative stress in a specific cellular compartment, the genetically encoded photosensitizer KillerRed was targeted either to the plasma membrane or to the histone protein H2B in the nucleus. Figure 3 shows fluorescence signals of KillerRed in these localizations (emitting at 610 nm), overlapped with fluorescence of molecular rotor BODIPY2 (at 520 nm) localized in the cell plasma membrane. For nuclear localization (H2B) of a phototoxic protein, the rotor signal in the membrane and the photosensitizer signal in the nucleus can be clearly distinguished. For plasma membrane localization of KillerRed (PM), the signals show a significant overlap (Figure 3, merged column). The Manders colocalization coefficient was 0.842.

Irradiation of HeLa cells expressing KillerRed at 593 nm, 50 mW/cm^2^, and 75 J/cm^2^ for 23 min resulted in the photobleaching of the protein by ~45% in the case of its nuclear localization and by ~55% in the case of membrane localization, indirectly indicating the occurrence of a photodynamic reaction.

**Figure 4:**
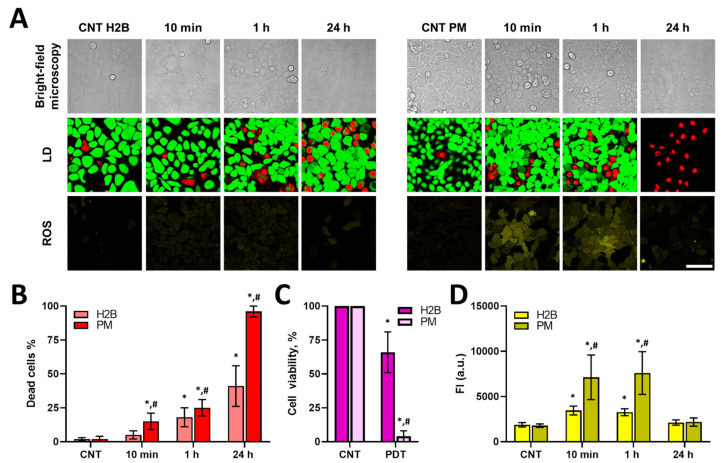
Cell viability and ROS analysis in cells after PDT with genetically encoded KillerRed protein. (**A**) Live (green)/dead (red) cells (LD) and ROS assay fluorescence images 10 min and 1 h after PDT. The bar is 40 µm, applicable to all images. (**B**) Quantitative analysis of dead cells in control and treated cell populations, %. (**C**) Viability of control and treated cells in 24 h after PDT, determined using the MTT assay. (**D**) Quantitative analysis of ROS in control and treated cell populations. Fluorescence intensity of DCFH-DA is shown as means ± SD. CNT: control with different localization of KillerRed. H2B: cells with nuclear localization of KillerRed. PM: cells with membrane localization of KillerRed. * *p* < 0.05 with control; # *p* < 0.05 with KillerRed-H2B.

Despite a similar photobleaching rate for the two localizations of KillerRed, the intracellular level of ROS detected by the DCFH-DA dye was different (Figure 4C). In the case of KillerRed-H2B, ROS production had almost doubled at 10 min after PDT and remained at the same level after 1 h. In the case of KillerRed-PM, the amount of ROS was ~3.5 times higher than in the untreated control in the time period of 10 min to 1 h. At 24 h after PDT, the amount of ROS was comparable with the control for both localizations of KillerRed. Using this ROS detection method, we cannot decouple different rates of ROS production from different rates of local ROS reactions. Therefore, assuming KillerRed produced the same concentration of ROS under identical irradiation conditions at these two locations, one possible explanation for the higher concentration of ROS detected with DCFH-DA for KillerRed-PM is a higher concentration of oxygen within lipid bilayers [19]. A ready attack of ROS on histones and DNA, which are close to the phototoxic protein, and the presence of various antioxidant enzymes and molecules in nuclei, which can neutralize ROS, may also lead to lower ROS generation in the case of KillerRed-H2B [20,21].

Cell viability assays revealed that cells with membrane-localized KillerRed died faster compared to ones with nuclei-localized KillerRed (Figure 4B). In the case of KillerRed-PM, the number of dead cells increased from 2% to 15% in 10 min after irradiation and increased to 25% (*p* = 0.00001) within 1 h after PDT. After 24 h of PDT, almost all cells were dead. In the case of KillerRed-H2B, the number of dead cells reached 18% at the 1 h mark and 41% at 24 h (*p* = 0.00007). In addition, viability analysis using the MTT assay showed that the proportion of viable cells decreased to 66% after PDT with KillerRed-H2B and to 4% after PDT with KillerRed-PM.

Therefore, PDT with a photosensitizer localized within the plasma membrane was more effective than with the same photosensitizer in the chromatin at identical irradiation conditions. This was confirmed by our cell survival assays and was consistent with ROS assays.

### 3.2. Lipid Peroxidation After PDT with KillerRed

It is known that double bonds in unsaturated fatty acids, which are part of membrane lipids, are susceptible to oxidation, which is one of the key effects of PDT [22]. To verify that PDT with KillerRed induced lipid damage and peroxidation, its products—diene (DC) and triene (TC) conjugates—as well as Schiff bases (SB) were measured in the heptane and isopropanol phases, attributed correspondingly to the neutral and charged lipids extracted from the cells. Neutral lipids are mainly composed of triacylglycerols and wax esters, whereas polar lipids are primarily membrane glycolipids and phospholipids. The heptane phase releases neutral lipids, which are thought to make up lipid droplets and also play an important role in preventing lipotoxicity and oxidative stress. In the isopropanol phase, charged lipids are released and mainly included in the membranes, including organelle membranes.

**Figure 5:**
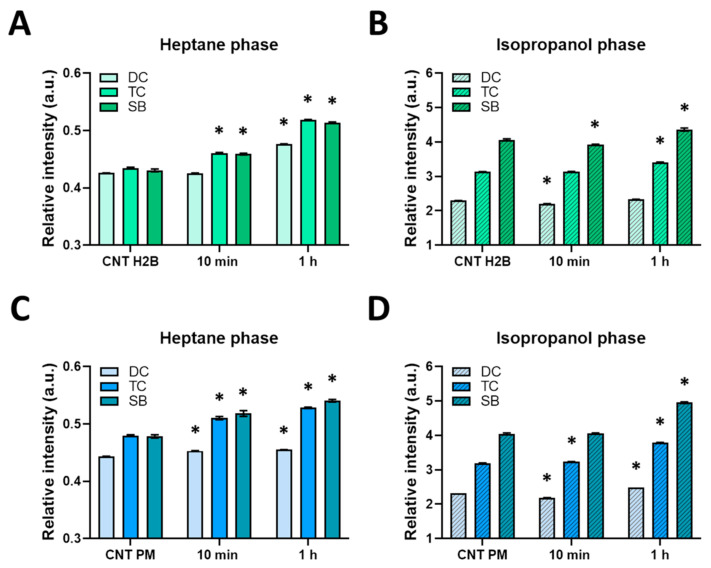
Analysis of products of lipid peroxidation: diene (DC) and triene (TC) conjugates and the Schiff bases (SB) for nuclear (**A**,**B**) and membrane localizations (**C**,**D**) of KillerRed. Means ± SD, *n* = 5 measurements. * Statistically significant differences in untreated control with KillerRed (*p* < 0.05). CNT H2B: control with nuclear localization of KillerRed, CNT PM: control with membrane localization of KillerRed.

It has been established that PDT with KillerRed at both localizations caused lipid peroxidation. In 10 min after PDT, TC and SB signals increased in the heptane phase (neutral lipids) for both localizations of KillerRed. The most pronounced effects were detected at 1 h after PDT. In the heptane phase, the DC signal increased by 11%, TC by 19%, and SB by 19% after PDT with KillerRed-H2B relative to control cells (Figure 5A). In the isopropanol phase (charged lipids), the DC signal increased by 1.5%, TC by 8.5%, and SB by 7.5% relative to control cells (Figure 5B).

PDT with KillerRed-PM resulted in an increase in the DC signal in the heptane phase by 3%, TC by 10%, and SB by 13% in 10 min irradiation (Figure 5C). The level of peroxidation products remained high at 1 h after PDT. A statistically significant increase in all three components was recorded in the isopropanol phase 1 h after PDT: the DC signal increased by 7%, TC by 19%, and SB by 23% (Figure 5D).

Interestingly, the local photosensitizer affected lipid peroxidation products. Thus, the greatest changes relative to control were recorded for neutral lipids when using KillerRed for nuclear localization and for charged lipids if peroxidation was initiated by KillerRed localized within the plasma membrane.

### 3.3. Plasma Membrane Microviscosity Changes After PDT in Monolayer Cells

The plasma membrane is the outermost boundary of each cell, and its fluidity and permeability are crucially important for rates of cell migration, ingress of chemicals, and protein interactions inside cells. We used fluorescence lifetime imaging microscopy (FLIM) in combination with a BODIPY2 molecular rotor, which was previously shown to selectively stain the plasma membrane of various cell lines [17,23]. Its lifetime can be directly correlated with microviscosity and lipid packing [24].

**Figure 6:**
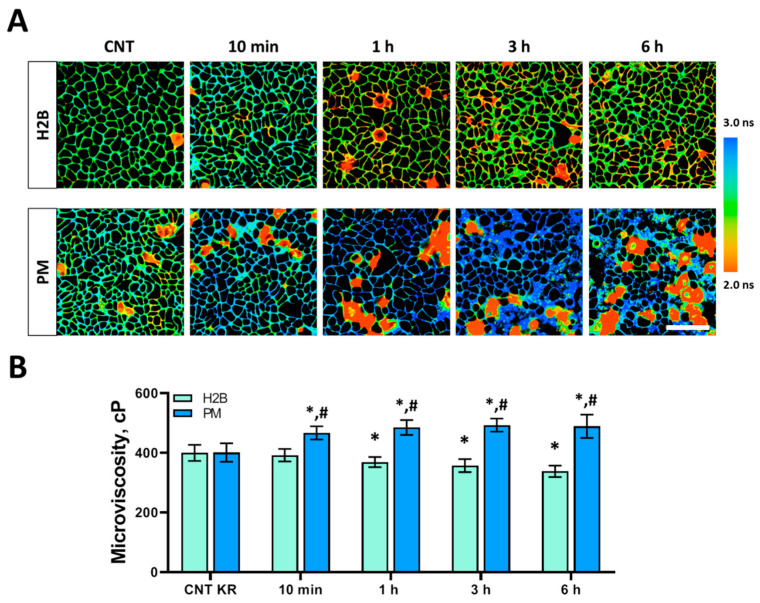
Plasma membrane viscosity in HeLa Kyoto cells with KillerRed during PDT. (**A**) Representative FLIM images of cells with both localizations of KillerRed. The bar is 40 µm, applicable to all images. (**B**) Quantification of viscosity of plasma membranes in HeLa Kyoto cells. Means ± SD, *n* = 100 cells for each time point. * *p* < 0.05 with control; # *p* < 0.05 with KillerRed-H2B. CNT KR: control with different localization of KillerRed. H2B: cells with nuclear localization of KillerRed. PM: cells with membrane localization of KillerRed.

We recorded the FLIM of BODIPY2 Hela Kyoto cells after PDT with the phototoxic protein KillerRed localized either in the nucleus or in the plasma membrane (Figure 6A). In control cells expressing KillerRed, the fluorescence decay time of the rotor in the plasma membrane was ~2.88 ns, which corresponded to a viscosity value of 400 cP irrespective of the localization of KillerRed.

Following continuous irradiation inducing PDT and cell death (Figure 3), viscosity was measured. Interestingly, the effects of PDT on the microviscosity of plasma membranes of cells were different for different localizations of the photosensitizer KillerRed (Figure 6). In cells with KillerRed-H2B, microviscosity gradually decreased to 369 ± 17 cP (*p* = 0.0009) at 1 h after PDT and then to 338 ± 19 cP (*p* = 0.0006) at 6 h.

For cells with membrane localization of the photosensitizer KillerRed-PM, a dramatic increase in membrane microviscosity was initially recorded. In the first minutes after PDT, microviscosity increased from 405 ± 31 cP to 467 ± 22 cP (*p* = 0.0007) and continued to increase. At 6 h post-PDT, the value was 489 ± 39 cP (*p* = 0.0005). However, the cells were not viable 24 h after PDT, causing BODIPY2 internalization, so microviscosity measurements were not performed in this case.

### 3.4. Changes in Membrane Microviscosity After PDT in 3D Spheroid Cells

Next, we examined the effects of PDT on the membrane microviscosity of three-dimensional tumor spheroids. This in vitro tumor model is more intricate than monolayer cells, as it better mimics cell–cell interactions and tumor heterogeneity. Control untreated spheroids were represented by structures with a dense core 200–250 μm in diameter.

**Figure 7:**
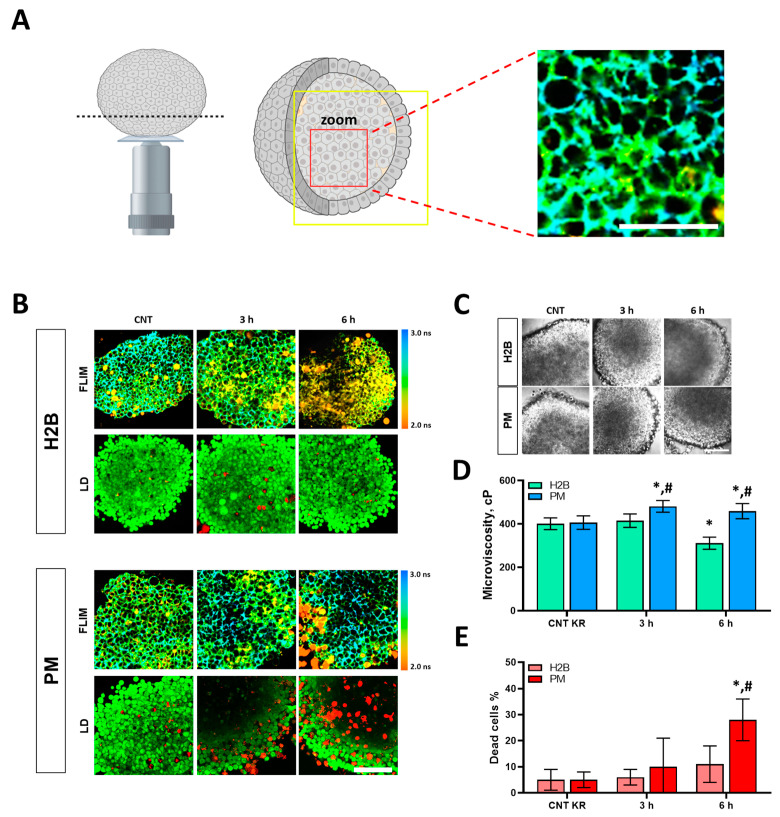
Plasma membrane microviscosity in HeLa tumor spheroids after PDT with KillerRed localized in the nuclei (H2B) or within the plasma membrane (PM). (**A**) Schematic representation of the spheroid area (shown by the yellow square) imaged by FLIM. The spheroid had adhered to the glass bottom, and the images were acquired from a depth of ~30 μm. Higher-magnification image of the molecular rotor distribution in spheroid cell membranes indicated by the red squares. The scale bar is 80 μm. (**B**) FLIM images and live/dead (LD) assay of control and treated cells in spheroids. Bar = 80 μm. (**C**) Morphology of control and treated spheroids. The scale bar is 80 μm. (**D**) Quantification of membrane microviscosity of spheroid cells after PDT. Means ± SD, *n* = 4 spheroids, 60 cells in each. (**E**) Quantitative analysis of dead cells in control and treated cell populations, %. * *p* < 0.05 with control; # *p* < 0.05 with KillerRed-H2B. CNT KR: control with different localization of KillerRed. H2B: cells with nuclear localization of KillerRed. PM: cells with membrane localization of KillerRed.

As was the case for 2D cell cultures, we saw significantly higher phototoxicity for membrane-localized KillerRed compared to nuclear Killer Red (Figure 7B).

After PDT with KillerRed-H2B, membrane microviscosity in tumor spheroids was lower than in the control starting from 6 h: 311 ± 28 cP (*p* = 0.00009) (Figure 7D). In addition, spheroids became loose with the disappearance of the core and the decrease in cell density in spheroids. The number of dead cells increased from 5% to 11% by 6 h after PDT (Figure 7E).

In the case of KillerRed-PM, membrane microviscosity increased dramatically from 406 ± 31 cP to 481 ± 27 cP (*p* = 0.00001) at 3 h and remained high for 6 h after PDT (Figure 7D). The number of dead cells had increased from 4% to 28% by 6 h after PDT (Figure 7E). Therefore, changes in membrane microviscosity induced by PDT with genetically encoded photosensitizer KillerRed were similar for cells in both 2D (monolayer) and 3D (tumor-like spheroids) structures, although the dynamic of changes was slower in the case of spheroids.

At this point, we can only speculate about the differential microviscosity trends observed in membrane vs. nuclear-targeted photosensitizers, because we did not collect the lipidomic data, e.g., on peroxidized products, that could confirm our hypothesis. However, according to the previous studies in model systems, decreased microviscosity of the membrane (seen upon irradiation of KillerRed in the nucleus) corresponds to radical products’ production, while increased microviscosity (seen upon the use of KillerRed in the plasma membrane) indicates the production of peroxides.

## 4. Discussion

One of the major effects of PDT at the cellular level is the oxidation of lipids constituting the membranes. PDT-induced cell death due to destruction of membranes is mediated by alterations in their physical state, which, however, is insufficiently investigated at present.

Here, we demonstrated that the early effects of PDT on the microviscosity of plasma membranes of cancer cells depend on the subcellular localization of the photosensitizer, which has not been shown previously.

The specific targeting of the photosensitizer and thus the induction of the oxidative stress exclusively within a specific cellular compartment became possible with the use of the genetically encoded phototoxic protein KillerRed. KillerRed is a red dimeric fluorescent protein that acts as a type I photosensitizer of Type I. The ability of KillerRed to kill cancer cells upon irradiation with light, both in cellulo and in vivo, was reliably established in previous works by our group and others [14,18]. It was found earlier that KillerRed at plasma membrane causes mainly necrosis of cells [15,16,25], while KillerRed localized in chromatin induces damage of DNA, activation of reparation machinery, and blockage of cell division [26,27].

Different effects of PDT on membrane microviscosity have been described, which was mainly associated with the type of photoreaction (electron transfer or singlet oxygen-based) and consequently with different products reacting with lipids. The major mechanism for the increase in viscosity was proposed to be peroxidation of lipids by singlet oxygen following type II reactions. There are several studies that have demonstrated an increase in viscosity/decrease in fluidity of lipid membranes as a result of lipid peroxidation during PDT. For instance, Paez-Perez et al., using FLIM with molecular rotors, detected higher microviscosity in model membranes consisting of peroxidized lipids compared to non-oxidized lipids. The polar hydroperoxide functional group -OOH from peroxidized lipid increased molecular ordering within the membrane and the lateral heterogeneity of the bilayer with the appearance of more ordered lipid clusters [28]. Vyšniauskas et al. recorded an increase in viscosity of lipid bilayer in a model of giant unilamellar vesicles (GUVs) in the presence of three different porphyrin-based photosensitizers [7]. Lakos et al. observed, using fluorescence anisotropy measurements, a decrease in membrane fluidity in mouse myeloma cells and phosphatidylcholine liposomes with membrane-bound hematoporphyrin as a photosensitizer [29]. Our recent research revealed, using FLIM, that PDT with the chlorine e6-based photosensitizer Photoditazine caused increased microviscosity in plasma membrane in cultured cancer cells and tumor xenografts [30]. Other studies have reported on the increase in local microviscosity in the cell cytoplasm during PDT. For example, with the use of a porphyrin dimer-based ratiometric rotor, Kuimova et al. demonstrated significantly increased intracellular viscosity during photoinduced cell death [9]. In a study by Izquierdo et al., PDT in cells using cyano-aryl porphyrazine was accompanied by a significant viscosity increase [10]. Higher microviscosity was also observed in mitochondria during PDT with mitochondria-targeting photosensitizers [31]. In the case of membrane localization of KillerRed, we observed a pronounced increase in membrane microviscosity in monolayer cells in the period from 10 min to 6 h after PDT. Thus, our results with membrane-targeting KillerRed-PM are consistent with these studies in terms of the effect on viscosity. Interestingly, the increased microviscosity was previously associated with type II photosensitizers, i.e., a direct reaction of singlet oxygen ^1^O_2_ with unsaturated lipids, producing peroxidation products. However, in a type I reaction, which is more probable for KillerRed, lipid hydroperoxide can also be generated with hydroxyl radical HO· as the proximal lipid oxidant [32]. Therefore, a strong interaction between a membrane-bound KillerRed and membrane lipids likely favored photoinduced peroxidation.

Decreased microviscosity of a lipid bilayer after PDT was observed for a type I oxidation process [6]. As shown in ref. [7], using methylene blue as a photosensitizer in a GUV model, decreased microviscosity of a lipid bilayer after PDT can be a result of cleavage of the double bond in lipid molecules. In addition, lipid hydroperoxides can be transformed into short-chain compounds (e.g., aldehydes) [33], the presence of which in the membrane can result in the formation of hydrophilic pores at moderate oxidative stress or in membrane disruption upon massive oxidation [34].

In our study with histone-targeting KillerRed-H2B, lower microviscosity of plasma membrane was detected starting from 1 h post-PDT in monolayer cells and from 3 h in spheroids. Since KillerRed is a type I photosensitizer, these results seem to be consistent with radical-induced cleavage. However, the principal difference of our present study is that a photosensitizer was not localized in the membrane in our experiments. It is known that ROS have an extremely short lifetime and a limited diffusion distance [35]. ROS generated by KillerRed in the nucleus can interact with different molecules in the cell before reaching the plasma membrane, giving rise to a large number of oxidation products and free radicals. Moreover, different antioxidant systems present in the cells can prevent oxidation processes through the removal of ROS [36]. Given the complexity of ROS-induced reactions in a complex biological environment, it is difficult to make any assumptions regarding the mechanisms that led to a fluidification of the plasma membrane after PDT with KillerRed-H2B.

In our previous work, we have investigated alterations of plasma membrane microviscosity induced by cytotoxic chemotherapy, which targets DNA and interferes with the cell division process [37,38], and in this sense is similar to PDT with KillerRed-H2B. However, during chemotherapy, the changes in microviscosity were more delayed (e.g., with cisplatin, oxaliplatin) or transient (e.g., with 5-fluorouracil). As such, the dynamics of viscosity changes observed after PDT with KillerRed-H2B allow us to assume that they resulted from ROS or radical attack to lipids rather than from adjustment in lipid composition in response to DNA damage.

If we compare the efficacy of PDT with KillerRed-H2B and KillerRed-PM, the former was more effective based on cell viability tests, which indicates the importance of the plasma membrane as a target for oxidative attack. According to our data, there was 2.5-fold higher percentage of cell death after PDT using membrane localization of KillerRed, and the changes in membrane microviscosity appeared earlier than with KillerRed-H2B. This means that an early increase in microviscosity of the plasma membrane due to severe ROS attack on it has more pronounced biological consequences in cancer cells than its delayed decrease mediated by nucleus damage. Although membrane fluidification has been previously associated with initiation of apoptosis via the Fas receptor [39], the efficacy of PDT with KillerRed-H2B is in general inferior to KillerRed-PM. Therefore, targeting PDT to the plasma membrane could be a more effective therapeutic strategy than targeting the nucleus.

Although oxidation of lipids is considered the major causative factor of microviscosity changes after PDT, an alternative pathway can be alterations in membrane lipid composition. Specifically, peroxidized lipids promote the formation of cholesterol domains and lipid rafts [40,41]. Cholesterol in turn is an important regulator of membrane viscosity that makes it more rigid [42]. In our previous study, lipidomic analysis revealed multiple changes in lipid profiles of cancer cells’ membranes after PDT with Photoditazine: a decrease in the amount of phosphatidylcholine and monounsaturated fatty acids and an increase in cholesterol and sphingomyelin [30].

A limitation of our study is that the experiments were conducted with only one cell type—HeLa Kyoto—for which stable cell lines expressing KillerRed were available. PDT is widely used to treat cervical cancer, so the choice of HeLa cells as a model system is relevant. Typically, the patterns of PDT effects are similar in different cell types and in vivo, which we observed earlier in our studies of membrane microviscosity upon PDT and chemotherapy [30,37,38]. A limitation of in vivo measurements of viscosity using FLIM and a BODIPY2 rotor is the diffuse nature of the rotor distribution in tumor cells within the tissue. As a result, different cellular structures may contribute to the microviscosity values measured in vivo, not only the plasma membrane. Therefore, it would be difficult to correlate the viscosity data with localization of the photosensitizer. KillerRed is applicable in vivo [18,26], although its current use in vivo was outside the scope of this study. We believe that the results obtained in the framework of the present research will be reproducible in other cell types and tumors, but this requires further verification.

## 5. Conclusions

Damage to the lipid bilayer is an important mechanism of PDT that has multiple consequences for cell functions, including those associated with permeability and diffusion and more complex signaling cascades. Irrespective of the localization of the photosensitizer in cancer cells, the plasma membrane becomes involved in the photooxidative reactions that modify its physical state. Our study has revealed that the microviscosity of plasma membranes changes in different directions when the same photosensitizer is located in the plasma membrane or in the nucleus. Direct interaction of ROS produced by membrane-targeted KillerRed (presumably, the superoxide anion radical) with lipids in the plasma membrane caused the increase in membrane viscosity, whereas the initial production of ROS in the nucleus was followed by a decrease in viscosity of the cell membrane. Based on these results, we can conclude that targeting plasma membranes is a promising strategy to kill cancer cells by PDT. The fact that KillerRed is less phototoxic than traditional chemical photosensitizers only highlights the promise of the PDT approach aimed at local oxidative destruction of cellular plasma membranes. These findings contribute to a better understanding of the effects of oxidative stress on lipid membranes and can guide future development of novel photosensitizers. Our study is mechanistic in nature; however, it represents an important first step in improving therapeutic outcomes in the future.

## Data Availability

All data related to the study can be provided by the corresponding author upon reasonable request.
